# Analysis of microRNA-transcript regulatory networks in the hippocampus of the BTBR mouse model of autism

**DOI:** 10.3389/fncel.2025.1676316

**Published:** 2025-10-27

**Authors:** Silvia Gasparini, Valerio Licursi, Arianna Rinaldi, Laura Ricceri, Maria Luisa Scattoni, Carlo Presutti, Cecilia Mannironi

**Affiliations:** 1 National Research Council, Institute of Molecular Biology and Pathology, Rome, Italy; 2 Department of Biology and Biotechnology “C. Darwin”, Sapienza University of Rome, Rome, Italy; 3 Center for Research in Neurobiology “D. Bovet”, Rome, Italy; 4 National Institute of Health, Rome, Italy

**Keywords:** microRNA, transcriptome, BTBR, autism, hippocampus, post-transcriptional regulation of gene expression, TDMD

## Abstract

Autism spectrum disorder (ASD) is a heterogeneous neurodevelopmental condition with unknown etiology. Currently, the role of post-transcriptional mechanisms in ASD remains unclear. microRNAs (miRNAs) are small non-coding regulatory RNAs that mediate mRNA destabilization and/or translational repression. To investigate the potential role of miRNAs in ASD, we performed miRNA expression profiling in the hippocampus of the BTBR ASD mouse model and age-matched C57BL/6 J mice. Alongside, we analyzed the BTBR hippocampal transcriptomic profile to identify differentially expressed transcripts (DETs). By integrating differentially expressed miRNA (DEmiRNA) and DET lists, we discovered mRNA transcripts that are putative targets of BTBR DEmiRNAs and exhibit an anti-correlated differential expression in the BTBR hippocampus. These interactions suggest potential regulatory networks related to gene transcription regulation, and synaptic structure and function relevant for ASD. These include miR-200 family members, miR-200a-3p, miR-200b-3p, miR-200c-3p, and miR-429, and the experimentally validated target, the transcription factor Zeb2. Moreover, we identified a set of non-canonical interactions characterized by extensive pairing between BTBR DEmiRNAs and DETs, potentially triggering target-directed miRNA degradation (TDMD). Our findings support a role for miRNA dysregulation in the pathophysiology of ASD.

## Introduction

Autism spectrum disorder (ASD) is a behaviorally defined neurodevelopmental disorder whose prevalence has dramatically increased during the past two decades: about 1 in 150 8-year-old children in 2000, to 1 in 31 in 2025 in the United States (Hyman et al., 2020; Maenner, 2023; Shaw et al., 2025). Core symptoms are identified in two main domains: social communication/interaction and restricted, repetitive patterns of behavior (Bell, 1994). ASD is characterized by phenotypic and genetic heterogeneity. To date, the exact disease mechanism remains unknown. Hundreds of genes have been implicated, but only a small fraction of them have sufficient genetic evidence to be considered causative (Geschwind, 2011). Recently, it has been emphasized that many of them converge on molecular pathways involved in activity-dependent signaling related to synapse development and plasticity (Geschwind, 2008; Ebert and Greenberg, 2013). A widespread dysregulation of brain gene expression has been observed in experimental and human ASD (Daimon et al., 2015; Parikshak et al., 2016; Gasparini et al., 2020; Satterstrom et al., 2020a; Wang et al., 2020; Mooney et al., 2025). Emerging evidence indicates that altered post-transcriptional mechanisms of gene expression regulation may strongly contribute to the pathophysiology of ASD (Wu et al., 2016; Gasparini et al., 2020; Dominguez-Alonso et al., 2023; Mooney et al., 2025; Yao et al., 2025). In the nervous system, complex post-transcriptional mechanisms, such as RNA splicing, mRNA stability and translation, tightly control normal cellular function (Cao et al., 2006). Different classes of non-coding RNAs (ncRNAs), including long non-coding RNAs (lncRNAs), circular RNAs (circRNAs), and microRNAs (miRNAs), represent important regulators of such mechanisms (Goldie and Cairns, 2012). Growing evidence shows significant ncRNA dysregulation in blood, postmortem brain tissues of individuals with ASD, and in the brain of ASD mouse models (Dominguez-Alonso et al., 2023). Specifically, the expression of some miRNAs is altered in experimental and human ASD (Wu et al., 2016; Frye et al., 2021; Mooney et al., 2025; Yao et al., 2025). miRNAs are small ncRNAs able to control the expression of hundreds of genes simultaneously, influencing cellular functions at the pathway level (Bartel, 2018). In the brain, they regulate processes that are pivotal to neuronal development and function, including neurogenesis, neuronal maturation, and synaptic plasticity (Saba and Schratt, 2010). Therefore, they are potentially very significant in the context of neurodevelopmental diseases, ASD dysregulated miRNAs representing potential biomarkers for diagnosis and targets for therapeutic intervention. We recently performed a genome-wide gene expression analysis of the hippocampus of the BTBR T + tf/J (BTBR) mouse model for idiopathic autism, an inbred mouse strain that incorporates multiple behavioral phenotypes relevant to all main diagnostic symptoms of autism (McFarlane et al., 2008). In this study we identified differentially expressed genes (DEGs) and circRNAs (DECs) (Gasparini et al., 2020). Here, we analyze the hippocampal miRNA and transcriptomic expression profiles of BTBR mice, finding differentially expressed miRNAs (DEmiRNAs) and transcripts (DETs). By integrating DEmiRNA and DET lists, we identified potential regulatory networks relevant to the disease. Finally, having identified BTBR DET and DEmiRNA non-canonical intermolecular interactions, we suggest novel mechanisms for miRNA turnover regulation in ASD.

## Materials and methods

### Animals

Subjects were adult male mice of the inbred strains BTBR T + Itpr3tf/J (BTBR) and C57BL/6 J (B6). Mice were purchased from the Jackson Laboratory (United States) and then bred and maintained in the vivarium of the National Institute of Health (Rome, Italy). Animals were housed in groups of three to five in standard cages and maintained at a constant temperature of 22 ± 1 °C on a 12-h light/dark cycle, with *ad libitum* access to food and water. All efforts were made to minimize animal suffering and to reduce the number of animals used. All procedures were in strict accordance with the EU Directive 2010/63/EU for animal experiments and the Italian Animal Welfare legislation (D.L. 26/2014). Mice were 12 weeks old and with matched body weights at the time of brain dissection. Mice were anaesthetized with isoflurane and rapidly decapitated. Hippocampi were dissected by punching of 1 mm brain slices. Samples from individual mice were collected in QIAzol (Qiagen), frozen with dry ice, and stored at −80 °C until processing.

### Tissue collection and RNA isolation

Total RNA was extracted from single mouse tissues, using QIAzol and miRNeasy spin column (Qiagen), with DNase1 on column-treatment, according to the manufacturer’s protocols (Qiagen). RNA concentration was determined by the NanoDrop 1,000 analysis (Thermo Scientific). RNA quality was assessed by gel electrophoresis and by measuring 260/280 and 260/230 absorbance ratios. RNA sequencing studies for miRNA and transcriptomic profiling were performed on RNA preparations from the same animal cohorts of BTBR and B6 mice.

### sRNA-seq

SmallRNA-seq (sRNA-seq) was performed on small RNA libraries obtained from hippocampal RNA samples from single animal preparations (BTBR *n* = 4, B6 *n* = 4, mice of the pool 2 cohort). sRNA-seq was performed on NextSeq500 using the Illumina TruSeq Small RNA Library Preparation Kit (Illumina, San Diego, United States). An average of 15 million reads per sample was obtained. The read quality was evaluated using FastQC (version 0.11.2; Babraham Institute, Cambridge, United Kingdom). After adapter cleaning, the resulting reads ranged from 94.7 to 97.7%. Reads were mapped to the mouse genome (Genome Reference Consortium mouse GRCm38) using the sRNAbench command line tool with ‘genome mapping mode’ from the sRNAtoolbox suite of software (Aparicio-Puerta et al., 2019). The sRNAbench pipeline uses Bowtie to align reads to the reference genome, then compares their coordinates to miRBase v21 annotations. Reads fully within reference RNA coordinates are assigned accordingly. Differential expression analysis of microRNAs is performed with the sRNAde pipeline from sRNAtoolbox. The module generates an expression matrix and uses the R/Bioconductor package edgeR (Robinson et al., 2010) to infer differential expression. By using edgeR, sRNAbench applies TMM normalization for the detection of differentially expressed microRNAs, which has been reported to be among the most stable methods.

### RNA-seq

RNA-seq was performed on two hippocampal RNA pools made from equal RNA amounts prepared from different animal cohorts of BTBR and B6 mice, pool 1 (*n* = 6) and pool 2 (*n* = 4). Library preparation was performed using the Illumina TruSeq Stranded Total RNA Library Preparation kit with Ribo-Zero treatment (Illumina, San Diego, United States) (Gasparini et al., 2020). Reads were mapped to the mouse Ensembl GRCm38 transcriptome index (release 84) using kallisto (version 0.42.5) (Bray et al., 2016). Transcript-level normalization and differential transcript expression analysis were performed using R/Bioconductor package tximport version 1.25.1 (Soneson et al., 2016) and DESeq2 version 1.26 (Love et al., 2014), accounting for the presence of batch effects.

### RT-qPCR

For miRNA quantitative analysis, 200 ng of RNA was reverse transcribed using the miRCURY LNA RT Kit (Qiagen), including an RT− (no enzyme) control reaction to check for residual DNA contamination. Quantitative PCR (qPCR) was performed using the miRCURY LNA SYBR Green PCR Kit (Qiagen) and the following miRCURY LNA miRNA PCR assays (Qiagen, Cat. No. 339306) with their GeneGlobe iDs: mmu-miR-429-3p, YP00205068; hsa-miR-200a-3p, YP00204707; hsa-miR-200b-3p, YP00206071; hsa-miR-200c-3p, YP00204482; hsa-miR-183-5p, YP00206030. For transcript quantitative analysis, RNA was reverse transcribed using SuperScript IV (Thermo Fisher Scientific) and random/dT primer mix, and qPCR performed using SYBR Green (SensiMix™SYBR Low-ROX Kit, Meridian Bioscience) with appropriate primers (Grin2aFwr: 5’ AAACGAGGTGGTCAGGTTCC 3′, Grin2aRev: 5’ CCATTTGCCACTCCCTGGAT 3′, Zeb2Fwr: 5’ GGCGAGCCAGAAAAGAAAA 3′, Zeb2Rev: 5’GAACAAAACCTCGCCAAGAG 3′, GapdhFwr: 5’ ACTTGAAGGGTGGAGCCAAA 3′, GapdhRev:5’ TCATGAGCCCTTCCACAATG 3′). RT-qPCR experiments were performed on the same RNA preparations used for sequencing analysis. Relative quantification of gene expression was conducted with the Applied Biosystems StepOnePlus RT-PCR System. U6 and Gapdh were used as internal controls, for miRNA and mRNA, respectively. RT-qPCR data were analyzed by the 2−ΔΔCt method (Livak and Schmittgen, 2001). BTBR and B6 were compared by an independent sample *t*-test, with significance set at *p* ≤ 0.05. RT-qPCR graphs were generated using GraphPad Prism 8.0.2.263.

### Bioinformatics analysis

To understand the biological meaning of the differentially expressed microRNAs, we performed enrichment analysis of predicted and experimentally validated microRNA/target interactions using the MIENTURNET webtool.^1^ To provide quantitative estimates of microRNA-mediated repression for both canonical and noncanonical site detection, microRNAs found as differentially expressed were evaluated with R/Bioconductor package scanMiR (Soutschek et al., 2022),^2^ which enables high-throughput prediction of their target sites on mRNA sequences. The package leverages experimentally determined binding affinity models to provide quantitative estimates of the binding affinity between a microRNA and a potential target site on an mRNA transcript, expressed as the dissociation constant (Kd) (Dillies et al., 2013). Log_kds in Supplementary Table S5 reports the ln(Kd) multiplied by 1,000, rounded and saved as an integer. DEmiRNA target and DET lists were overlapped with the SFARI genes downloaded from the SFARI database (1,230 genes, last released January 2025).^3^ The Venn diagram was generated using the ggVennDiagram Shiny app.^4^ Gene Ontology (GO) analyses were performed using the R package clusterProfiler. The simplify method was applied within the enrichGO function to remove redundant terms. A cutoff of 0.7 was used for the adjusted *p*-values (p.adj) in the GO analysis of DEmiRNA targets and the anticorrelated DET-DEmiRNA targets. The anticorrelated interacting network between 69 DETs and DEmiRNAs, as well as between Zeb2 and the upregulated DEmiRNAs, was generated with Cytoscape (Shannon et al., 2003).

### Statistical analysis

RT-qPCR data are shown as the mean ± standard error or deviation, for miRNA and Zeb2 quantitative analysis, respectively. Statistical significance was evaluated by a two-sided unpaired Student’s *t*-test, *performed on at least biological replicates and using the average value of technical replicates*. Spearman’s correlation between microRNA and transcript log_2_ fold changes was evaluated in the R environment with base *cor* function. Supplementary Figure S1 was created in the R environment with the package ggplot2 version 3.3.0.

## Results

**Figure 1:**
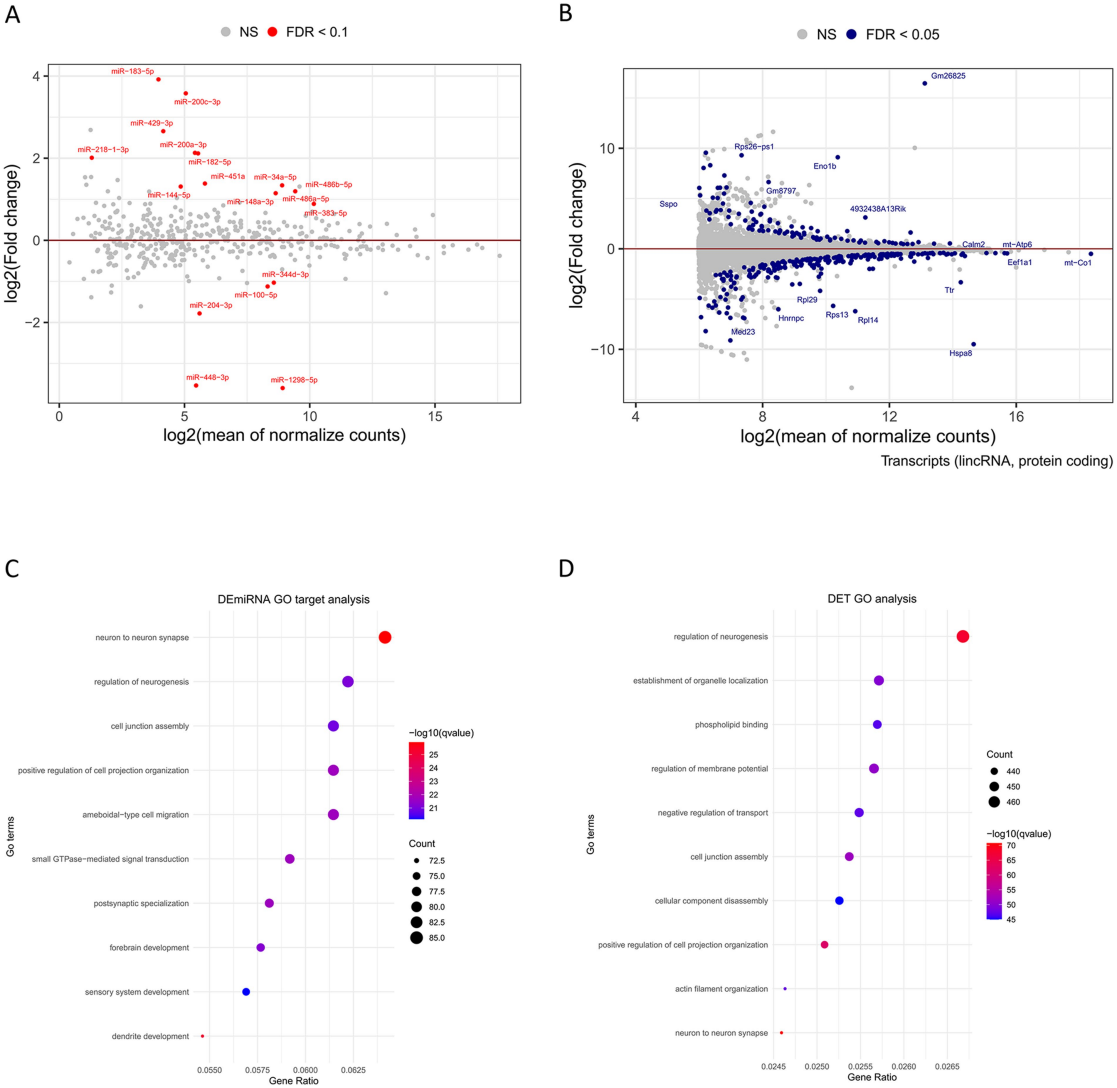
Differential expression of hippocampal miRNAs and transcripts in BTBR mice compared to B6 mice. MA plots represent log fold-change (base 2) versus mean expression between BTBR and B6 **(A)** miRNAs and **(B)** transcripts. Each dot on the graphs represents miRNA (red dots) or transcripts (orange dots) that are significantly differentially expressed in the hippocampus of BTBR vs. B6 mice, with upregulated above and downregulated below the continuous lines, respectively. Gray points represent non-significant changes. FDR < 0.1 is the statistical significance data cutoff. Gene Ontology (GO) analysis of BTBR **(C)** DEmiRNA target genes and **(D)** DETs according to Biological process, Cellular component, and Molecular function is reported.

In human ASD patients and mouse models, the hippocampus shows consistent abnormalities in neuronal morphology and cytoarchitectural organization (Varghese et al., 2017). To investigate the contribution of miRNA to ASD, we studied the hippocampal miRNA transcriptomic profile by RNA-Seq of small RNA libraries obtained from B6 control and BTBR mice. To date, the BTBR mouse strain is the most extensively characterized inbred strain for the core behavioral characteristics of ASD (Varghese et al., 2017). Our results revealed that 18 miRNAs are differentially expressed in BTBR compared to B6 mice, with 13 upregulated and five downregulated (Figure 1A; Supplementary Table S1). In parallel, to analyze at a transcript-level resolution the hippocampal transcriptomic profiles, we processed RNA-seq data from our previous study (Gasparini et al., 2020) to identify potential individual transcript isoforms differentially expressed in BTBR mice. Transcriptomic analysis, compared to gene expression analysis, provides a more detailed view of gene expression, as different isoforms of a gene may have different functions and regulation. In addition, as 3’UTRs might differ among transcript isoforms, transcriptomic analysis is required for a detailed miRNA/mRNA regulatory network investigation. Our analysis identified a total of 70,622 distinct transcripts, 536 of which were differentially expressed in the hippocampus of BTBR mice (Figure 1B; Supplementary Table S2).

To study BTBR DEmiRNA-mRNA target regulatory networks, we statistically analyzed a list of 18 DEmiRNAs selected using an FDR cutoff < 0.1 (Supplementary Table S1) list using the MIENTURNET web tool that identifies potential or experimentally validated miRNA gene targets, based on the TargetScan software^5^ and the MiRTarBase database^6^ (Licursi et al., 2019). TargetScan predicts biological targets of miRNA by searching for the presence of 8-6mer sites in the mRNA 3’UTR that match the seed region of the miRNA, according to canonical binding rules for miRNA-mediated mRNA degradation and translational repression (Bartel, 2018). MiRTarBase provides miRNA-target interactions validated by biological experiments. Our analysis revealed that 1,420 genes are potential targets of BTBR DEmiRNAs and 54 genes are validated targets (Supplementary Table S3).

**Figure 2:**
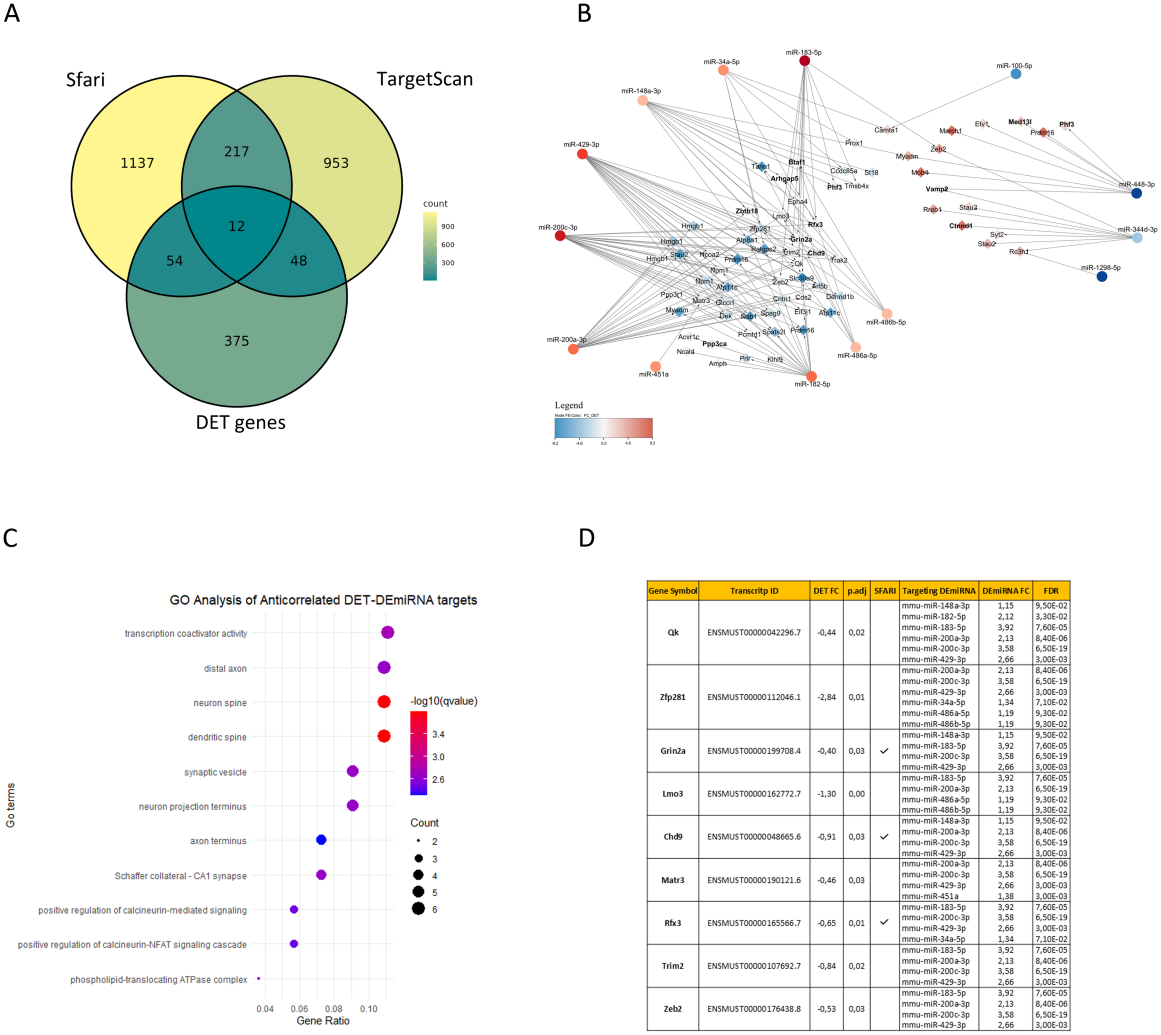
Interaction networks between BTBR hippocampal DEmiRNAs and DETs. **(A)** Venn diagram shows the overlap among BTBR hippocampal DEmiRNA target genes (1420), DET genes (489) identified in this study, and human SFARI genes (1230). **(B)** The interaction network displays DEmiRNA and DET putative targets with anticorrelated expression (69). Red and blue nodes represent upregulated and downregulated DEmiRNA (circles) or DETs (diamonds), respectively. In bold are SFARI genes. **(C)** Enriched ontology terms (GO) of anticorrelated DET putative targets; q-values and gene counts for each term are reported in the Figure. **(D)** The top anticorrelated interactions between DETs and DEmiRNAs are reported in the table with expression fold changes (FC) in BTBR vs. B6 hippocampi, with statistical significance (p.adj and FDR). Transcripts from SFARI genes are indicated.

Interestingly, our functional analysis of BTBR DEmiRNA predicted target genes and BTBR DETs revealed a convergence to enriched gene ontology pathways related to neurogenesis (GO:0050767), cell junction assembly (GO:0034329), cell communication (neuron to neuron synapse GO:0098984), and regulation of membrane potential (GO:0042391) (Figures 1C,D; Supplementary Table S6). To identify potential miRNA regulatory networks in the BTBR hippocampus, we integrated BTBR DEmiRNA target gene list with the DET list of 536 transcripts (FDR < 0.05, Supplementary Table S2), recognizing 82 DETs that are predicted targets of DEmiRNAs, according to TargetScan (Supplementary Table S4). We combined DEmiRNA target and DET lists with the SFARI gene list. SFARI is a curated database of ASD risk genes sourced from the Simons Foundation Autism Research Initiative (see footnote 3). This analysis revealed 12 SFARI genes that are BTBR DEmiRNA targets and that generate transcripts differentially expressed in the BTBR hippocampus. These genes are Arhgap5, Btaf1, Chd9, Ctnnd1, Grin2a, Med13l, Phf3, Ppp3ca, Ptprd, Rfx3, Vamp2, and Zbtb18 (Figure 2A; Supplementary Table S4).

**Figure 3:**
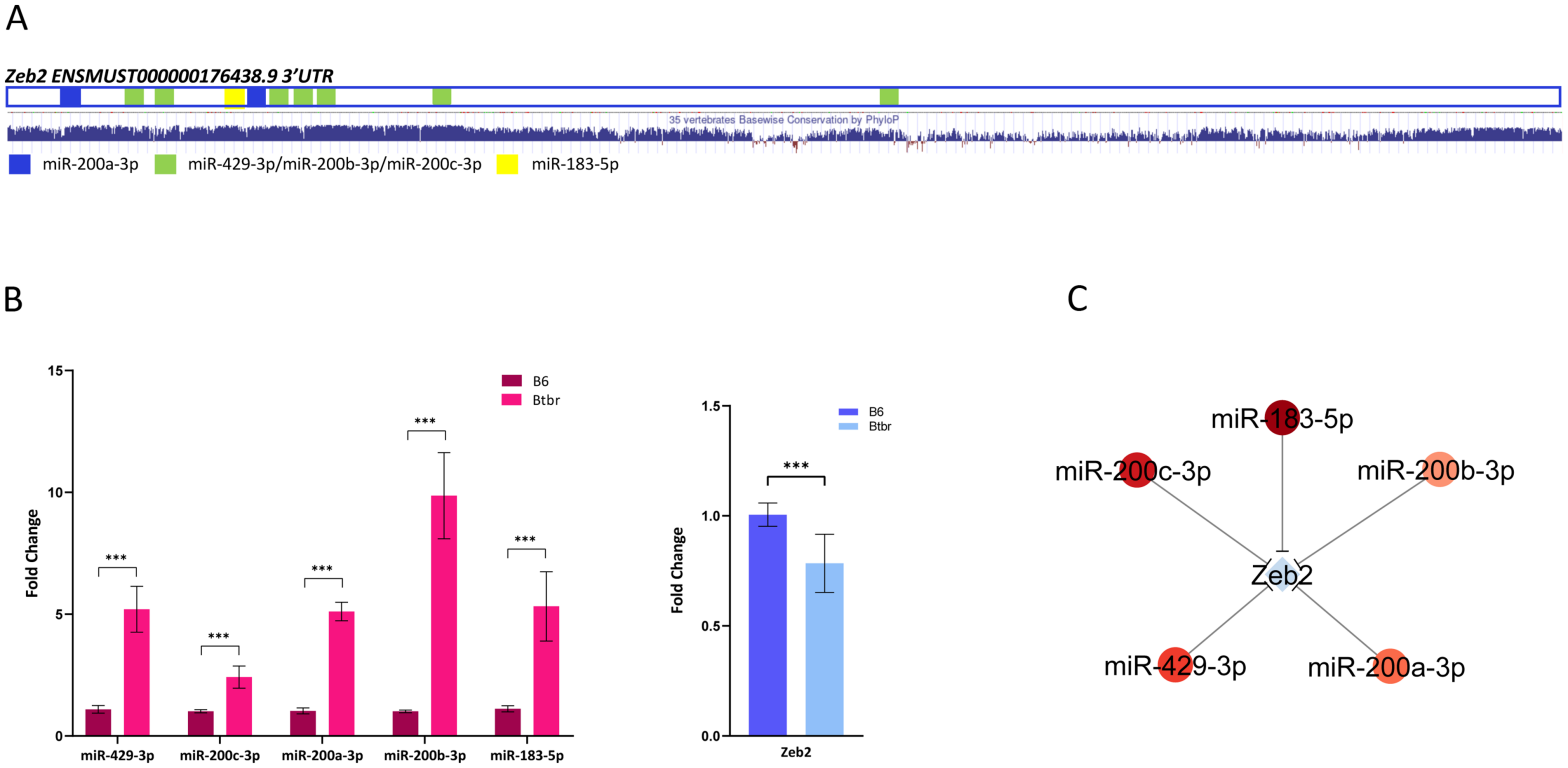
Zeb2 is a validated target of the BTBR DEmiRNAs. **(A)** The 3’UTR of the BTBR differentially expressed Zeb2 transcript (ENSMUST00000176438.8) with target sequences for BTBR up-regulated miRNAs is schematized. The PhyloP basewise conservation among vertebrates is reported below (https://genome.ucsc.edu/). **(B)** The expression of Zeb2 DET and targeting DEmiRNAs was validated by RT-qPCR on hippocampal RNA samples from single BTBR and B6 mice (*n* = 4 each group). Fold change is relative to B6 control mice. Data are expressed as mean ± SE, *n* = 3–6 technical replicates each (two-sided unpaired Student’s *t*-test was performed on biological replicates using mean values of technical replicates, ***p < 0.001). **(C)** Interaction network between Zeb2 transcript and BTBR DEmiRNAs.

Then, we identified among the 82 DETs that are predicted targets of DEmiRNA (Supplementary Table S4). Sixty-nine DETs, generated by 58 genes, showing anticorrelated expression with the targeting miRNAs (Figure 2B; Supplementary Table S4), which is significant accordingly to a Spearman’s correlation analysis (Supplementary Figure S1). This analysis revealed a total of 155 anticorrelated interactions, 133 involving 53 downregulated transcripts and 22 involving 16 upregulated transcripts (Figure 2B; Supplementary Table S4). Functional analysis performed on anticorrelated DETs identified enriched GO terms associated with transcription co-activation, neuronal signaling, and synaptic morphology and function (Figure 2C; Supplementary Table S6). To identify strongly anticorrelated interactions, we filtered for DEmiRNA targets recognized by at least two DEmiRNAs, identifying 128 strongly anticorrelated interactions involving 43 DETs: 124 interactions associated with 41 downregulated transcripts and 4 associated with 2 upregulated transcripts (Supplementary Table S4). Interestingly, among these mRNAs, we found eight SFARI genes (Arhgap5, Btaf1, Chd9, Grin2a, Phf3, Rfx3, Vamp2, and Zbtb18) (Figure 2B). Among these strongly anticorrelated DETs/DEmiRNAs interactions, we identified 9 transcripts (Qk, Zfp281, Grin2a, Lmo3, Chd9, Matr3, Rfx3, Trim2, and Zeb2) targeted by at least four DEmiRNAs (Figure 2D; Supplementary Table S4). Of those genes, 30% are SFARI genes (Grin2a, Chd9, and Rfx3) (Figure 2D). Grin2a reduced expression in the BTBR hippocampus was validated by RT-qPCR (Supplementary Figure S2). Zeb2 is the only experimentally validated target of anticorrelated DEmiRNAs, namely miR-200a-3p, miR-200c-3p, miR-183-5p, and miR-429-3p (Supplementary Table S3; Gregory et al., 2008). Of the two Zeb2 transcripts differentially expressed in our study (ENSMUST00000176438.8 and ENSMUST00000201804.3), ENSMUST00000176438.8 is the canonical Zeb2 mRNA, highly expressed in the mouse hippocampus, while ENSMUST00000201804.3 is a minor transcript variant weakly expressed in the hippocampus (Supplementary Table S2). From now on, our analysis will be focused on the ENSMUST00000176438.8 transcript. Zeb2 mRNA contains a total of 10 binding sites for the four anticorrelated DEmiRNAs (Figure 3A), suggesting a miRNA-mediated post-transcriptional regulation of the Zeb2 gene expression in the BTBR hippocampus. To validate relevant RNA-seq results, we performed RT-qPCR analysis of DEmiRNAs in the hippocampus of BTBR and B6 mice, proving significantly increased levels of miR-429-3p, miR-200a-3p, miR-200c-3p, and miR-183-5p in BTBR mice. As the expression of miR-200a-3p and miR-200b-3p is known to be tightly regulated (Korpal et al., 2008), by RT-qPCR we analyzed miR-200b-3p, although sRNA-seq did not detect significant changes (FDR = 0.12). Surprisingly, we found 10-fold higher levels of miR-200b-3p in the BTBR compared to B6 mice (Figure 3C). Similar discrepancies between RNA-seq and RT-qPCR results can occur due to differences in sensitivity and data normalization. In parallel, we confirmed that in the BTBR hippocampus the level of Zeb2 mRNA is significantly lower than in B6 mice.

**Figure 4:**
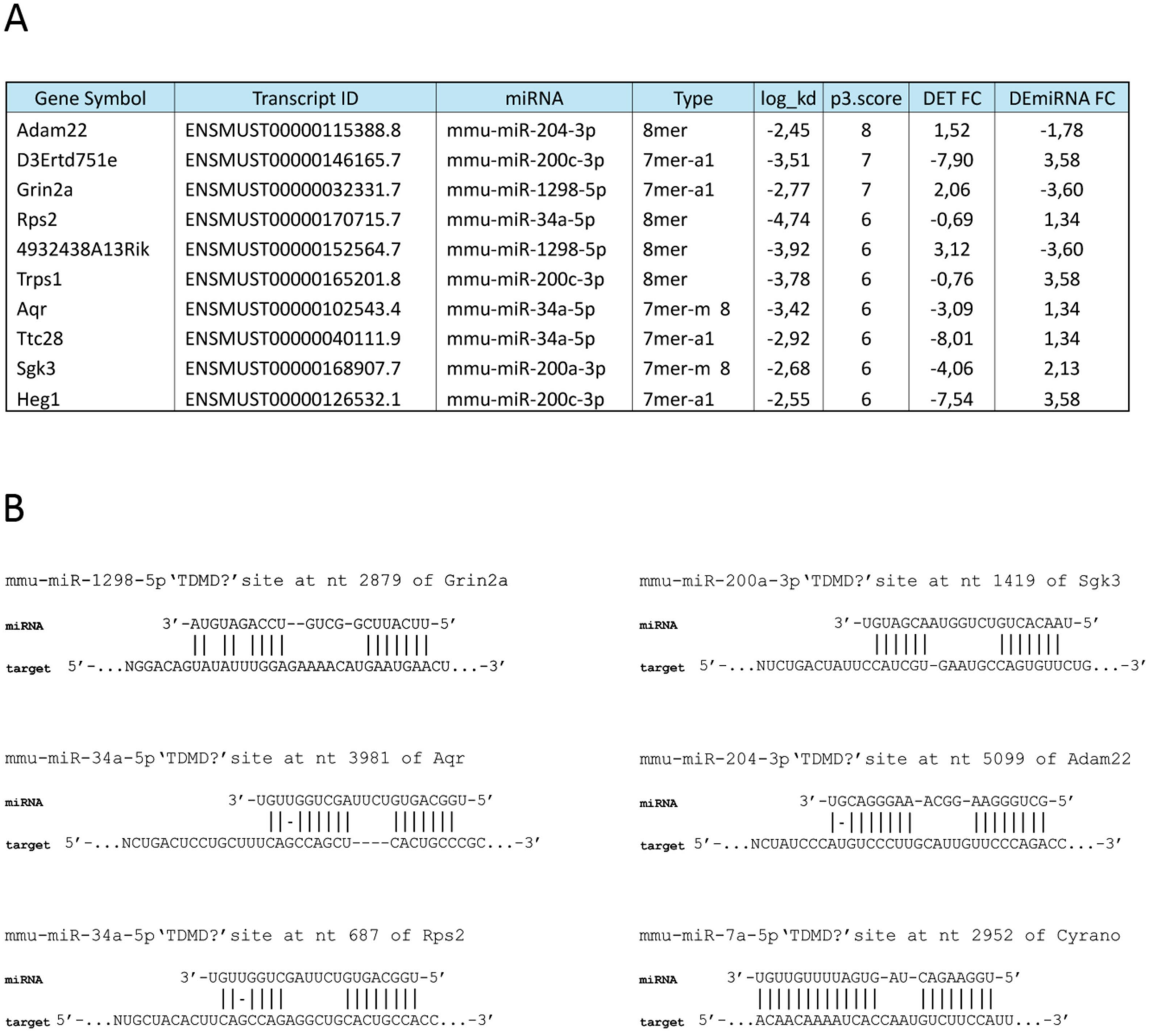
Non-canonical interactions between BTBR DEmiRNAs and DETs. **(A)** Identification of high affinity BTBR DEmiRNA and DET interactions potentially leading to TDMD, according to scanMiR package analysis. Interaction type, energy (log_kd) and scores (p3.score), together with miRNA and transcript FC are reported. **(B)** Examples of DEmiRNA/DET non-canonical bindings are reported, with miR-7a-5p/Cyrano interaction.

In the last few years, non-canonical miRNA/mRNA binding modalities have been described, characterized by extensive complementarity between the miRNA 3′ region and RNA targets, with a central bulge separating seed-matching (Hiers et al., 2024). This binding modality is sufficient to induce miRNA degradation in a regulated mechanism, named target-dependent miRNA degradation (TDMD). To identify potential TDMD interactions between BTBR DEmiRNAs and DETs, we processed our sRNA datasets using the scanMiR web package (Soutschek et al., 2022), which predicts unconventional interactions through the scanning of the input sequences. miRNA sites on target RNAs are classified as potentially inducing TDMD sites according to a greater complementarity of miRNA flanking nucleotides and the estimated dissociation rate constant (kd) (Soutschek et al., 2022). Our analysis revealed a total of 19 putative TDMD interactions, involving a total of nine DEmiRNAs and 18 DETs (Supplementary Table S5). Among these interactions, 10 exhibited anticorrelated expression (Figure 4A; Supplementary Table S5), with miR-200c-3p and miR-34a as the DEmiRNAs associated with the highest number of TDMD potential interactions. The top score interaction identified is between Adam22 mRNA and miR-204-3p, whose binding modality resembles the well-characterized TDMD-inducing interaction between Cyrano (Cdr1os) and miR-7a-5p (Figure 4B) (Kleaveland et al., 2018). It is worth noting that mir-204 has important implications in several neurodegenerative diseases (Tao et al., 2021) and regulates multiple Sfari genes (Chen et al., 2020). In conclusion, the identified high-affinity interactions between DEmiRNAs and target mRNAs suggest potential TDMD regulatory mechanisms of miRNA levels in the mouse hippocampus.

## Discussion

The present study provides the differential expression profile of miRNAs of the hippocampus of the BTBR mouse model for idiopathic autism. We identified 18 DEmiRNAs significantly deregulated compared to control B6 mice. Using computational predictions, we first assessed BTBR DEmiRNA putative target genes, based on the identification of binding interactions between seed regions and complementary sites on the mRNA 3’UTRs, known to determine translational repression and mRNA degradation. The integration of BTBR DEmiRNAs and DETs allowed us to identify anticorrelated miRNA and mRNA putative interactions enriched for GO terms relevant to ASD. Among such interactions, Zeb2 mRNA, recognized by five DEmiRNAs, is the only experimentally validated target of BTBR DEmiRNAs. Finally, the identification of high-energy non-canonical interactions between DEmiRNAs and DETs suggests a miRNA turnover regulation by TDMD mechanisms in the mouse hippocampus.

Recent research highlights the role of posttranscriptional regulatory mechanisms in ASD pathophysiology (Parikshak et al., 2016; Wu et al., 2016; Chen et al., 2020; Li et al., 2022; Dominguez-Alonso et al., 2023). miRNAs, which are small non-coding regulatory RNAs that mediate mRNA destabilization and translational repression (Bartel, 2018), are altered in human ASD samples and animal models (Wu et al., 2016; Mooney et al., 2025; Yao et al., 2025). Differential miRNA expression was observed in multiple studies performed in various ASD patient biofluids or tissues (Li et al., 2022; Garrido-Torres et al., 2024). Few high-throughput miRNA studies have been performed in ASD animal models (Garrido-Torres et al., 2024). In this regard, animal ASD models are fundamental to exploring the pathogenic mechanism of ASD *in vivo*. The BTBR inbred mouse strain is a well-characterized animal model of idiopathic ASDs that displays behaviors consistent with the diagnostic categories for ASD (McFarlane et al., 2008). BTBR miRNA expression profiles have been previously analyzed in the cortical brain and prefrontal cortex (Wang et al., 2020; Mooney et al., 2025). In this study, we performed an sRNA-seq analysis to characterize the miRNA profile in the hippocampus of BTBR mice, which has never been evaluated before. The BTBR hippocampus exhibits structural and functional alterations, and significant gene expression changes related to ASD dysfunctional pathways (McFarlane et al., 2008; Daimon et al., 2015; Gasparini et al., 2020). Our sRNA-seq analysis indicated a miRNA differential expression signature with 18 miRNAs strongly dysregulated. We hypothesize that the lack of overlap between DEmiRNAs identified in the hippocampus and those found in the studies by Wang et al. (2020) and Mooney et al. (2025) on BTBR brain cortical tissues, may be due to the spatially restricted or enriched expression and regulation of neuronal miRNAs in different anatomical regions (Cao et al., 2006). Some upregulated BTBR DEmiRNAs were previously associated with the ASD phenotype. miR-451a is one of the most frequently dysregulated miRNAs, identified in a recent meta-analysis performed across 16 published studies on ASD human patients (Garrido-Torres et al., 2024). Since miR-451 was associated with clinical manifestations of ASD, it may have relevance for clinical practice and experimental studies involving BTBR mice. miR-486 was previously associated with experimental and human ASD (Li et al., 2022; Garrido-Torres et al., 2024). miR-34a was previously identified as a repressor of the high-confidence Sfari gene Shank3 in mouse hippocampal neurons (Choi et al., 2015), and up-regulated in the cerebellum of the valproic acid ASD rat model (Dai et al., 2017). Interestingly, four out of 13 miRNAs significantly up-regulated in the BTBR hippocampus, miR-200a, miR-200b, miR-200c, and miR-429, belong to the miR-200 family (miRBase). miR-200 family members have a well-characterized role in the epithelial-mesenchymal transition (Gregory et al., 2008). Although miR-200 s are dysregulated in ASD (Ragusa et al., 2020), their role in the autistic pathophysiology has not been characterized yet. As endogenous miRNA target sites are found mainly in the 3’UTR of mRNAs (Gu et al., 2009), which might differ across multiple gene transcripts, to identify potential miRNA-mRNA interactions in our datasets, we analyzed the BTBR transcriptomic profile. Therefore, we processed RNA-seq raw data, which we previously obtained from the same mouse cohorts, to identify the transcriptomic differential expression profile at a transcript-level resolution. To identify potential regulatory networks involving BTBR DEmiRNAs, we selected inversely related DEmiRNAs-DETs pairs. This process allowed us to identify relevant regulatory miRNA/mRNA interactions that might be relevant for ASD. Functional analysis indicated a significant enrichment for terms related to transcription coactivator activity and components of neuronal communication. Dendritic spines, synaptic vesicles and axon terminals components were among the enriched terms related to neuronal communication, and they are implicated in activity-dependent signaling networks that control synapse development and plasticity. This finding is coherent with the growing evidence that many of the genes that are mutated in ASD are crucial components of the activity-dependent signaling networks that regulate synapse development and plasticity (Geschwind, 2008; Ebert and Greenberg, 2013). Interestingly, we identified strongly anticorrelated interactions between DETs and DEmiRNA, with DETs targeted by at least 4 DEmiRNAs. DET genes include Grin2a, Chd9, and Rfx3, which have SFARI scores of 1 or 3. GRIN2 is a member of the glutamate-gated ion channel protein family. GRIN2A variants have been found in patients with various neuropsychiatric disorders, including autism spectrum disorders, epilepsy, intellectual disability, attention-deficit/hyperactivity disorder, and schizophrenia (Barnby et al., 2005; Tarabeux et al., 2011). CHD9 is a chromodomain helicase DNA binding protein, a transcriptional coactivator, identified as an ASD risk gene (Satterstrom et al., 2020b). Rfx3 is a transcription factor playing a crucial role in different biological processes, including brain development. It was identified among disrupted genes in an exome sequencing study done in a large cohort of ASD patients (De Rubeis et al., 2014). Among strongly anticorrelated interactions found in the BTBR hippocampus, the downregulated Zeb2 mRNA is the only experimentally validated target of DEmiRNAs. Zeb2 is a zinc finger/homeodomain protein that functions as a DNA-binding transcriptional repressor. Mutations or deletions of Zeb2 cause the neurodevelopmental disorder Mowat-Wilson syndrome, a rare genetic disease characterized by features common to ASD (Hegarty et al., 2015). However, Zeb2 has never been directly associated with ASD. More experimental studies will be required to clarify its role in the disease. In the nervous system, miRNAs induce rapid and spatially localized changes in gene expression (Krol et al., 2010). Consistently, neuronal miRNA levels are tightly modulated in their biogenesis and stability (Cao et al., 2006). The recent discovery that the binding of highly complementary mRNAs or ncRNAs destabilizes miRNAs through the TDMD mechanism partially explains the highly dynamic regulation of neuronal miRNAs (Hiers et al., 2024). We then investigated potential regulation of miRNA level by TDMD mechanisms in the ASD mouse hippocampus. By the computational identification of high-energy interactions between anticorrelated DEmiRNAs and DETs we identified potential mRNAs able to induce miRNA degradation through a TDMD mechanism. Those interactions might represent an extra layer of miRNA regulation in the BTBR hippocampus. Further research will be needed to experimentally validate our predicted interactions. In conclusion, we provide strong evidence of a profound alteration of the miRNA profile in the hippocampus of BTBR mice. Integration analysis of BTBR DEmiRNAs and DETs pinpoints a downstream dysregulation of biological networks potentially relevant for ASD. In addition, our study offers some insights into a novel layer of miRNA regulation through molecular cross-talks. We believe that miRNAs may contribute to both genetic heterogeneity and phenotypic variation, representing promising novel targets for drug development in neurodevelopmental diseases.

## Data Availability

The sequencing datasets are publicly available at NCBI’s Gene Expression Omnibus (GEO) repository, under accession number GSE303513 located at https://www.ncbi.nlm.nih.gov/geo/query/acc.cgi?acc=GSE303513. Any additional information required to reanalyze the data reported in this work paper is available from the corresponding author upon request.

## Footnotes

^1^ http://userver.bio.uniroma1.it/apps/mienturnet/

^2^ https://ethz-ins.org/scanMiR/

^3^ https://gene.sfari.org/

^4^ https://bio-spring.shinyapps.io/

^5^ https://www.targetscan.org/mmu_72/

^6^ https://mirtarbase.cuhk.edu.cn/

## Supplementary material

SUPPLEMENTARY TABLE S1 BTBR DEmiRNAs.

SUPPLEMENTARY TABLE S2 BTBR DETs.

SUPPLEMENTARY TABLE S3 MIENTURNET analysis of BTBR DEmiRNAs.

SUPPLEMENTARY TABLE S4 Integration data analysis of anticorrelated DETs/DEmiRNAs.

SUPPLEMENTARY TABLE S5 scanMiR analysis of BTBR DETs/DEmiRNAs.

SUPPLEMENTARY TABLE S6 Gene ontology and functional analysis of DETs and DEmiRNAs.
