# Supplementary material for: Analysis of microRNA-transcript regulatory networks in the hippocampus of the BTBR mouse model of autism
Source: Front Cell Neurosci. 2025 Oct 27;19:1676316. doi: 10.3389/fncel.2025.1676316 (PMC12597957; doi:10.3389/fncel.2025.1676316)
Supplement: Supplementary file 7 [file Data_Sheet_1.pdf]

Figure S1

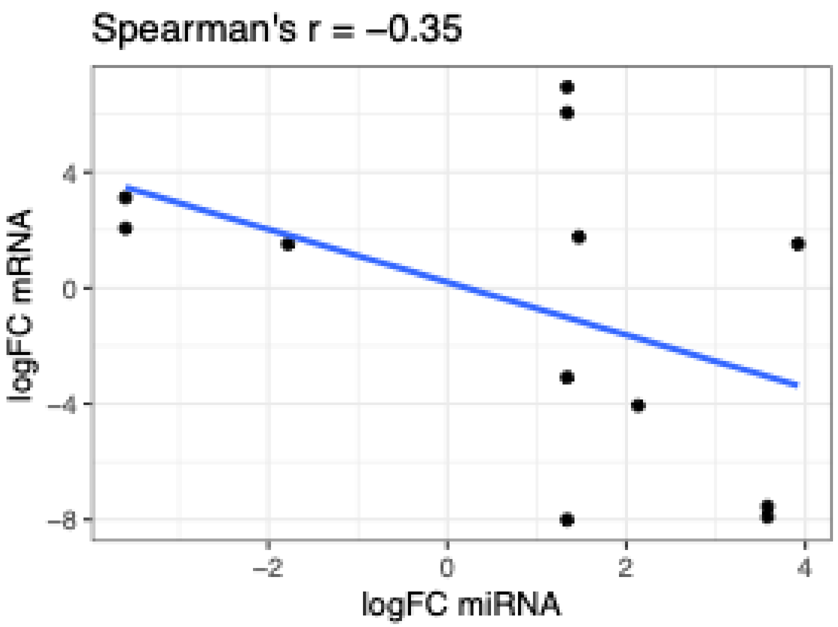

Figure S1. Spearman's correlation analysis of the logFCs of 18 DemiRNAs and 69 anticorrelated DETs (see Table S4)

Figure S2

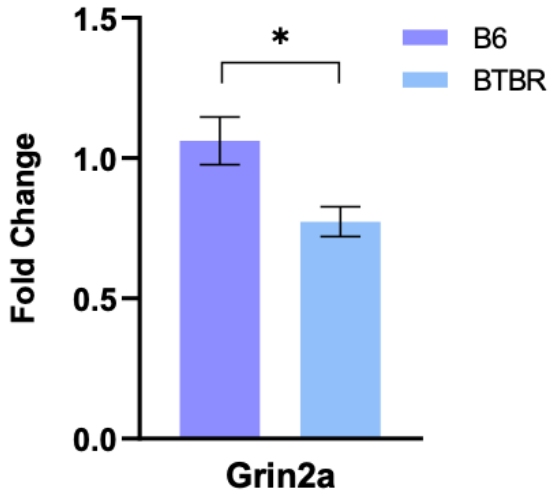

Figure S2. Validation of Grin2a downregulation in BTBR mouse hippocampus.
The expression of Grin2a DET was validated by RT-qPCR on hippocampal RNA samples from BTBR and B6 mice (n=4 each group). Fold change is relative to B6 control mice. Data are expressed as mean ± SE, n= 3–6 technical replicates each (two-sided unpaired Student's t-test, *p = 0.03).
