# Supplementary material for: Analysis of microRNA-transcript regulatory networks in the hippocampus of the BTBR mouse model of autism
Source: Front Cell Neurosci. 2025 Oct 27;19:1676316. doi: 10.3389/fncel.2025.1676316 (PMC12597957; doi:10.3389/fncel.2025.1676316)
Supplement: Supplementary file 8 [file Data_Sheet_2.pdf]

Supplementary Material

1 Supplementary Tables and Figures

1.1 Supplementary Tables

Supplementary Table S1: BTBR DEmiRNAs

Supplementary Table S2: BTBR DETs

Supplementary Table S3: MIENTURNET analysis of BTBR DEmiRNAs

Supplementary Table S4: Integration data analysis of anticorrelated DETs/DEmiRNAs

Supplementary Table S5: scanMiR analysis of BTBR DETs/DEmiRNAs

Supplementary Table S6: Gene ontology and functional analysis of DETs and DEmiRNAs

1.2 Supplementary Figures

Supplementary Figure 1. Spearman's correlation between miRNA and transcript expression changes.

Supplementary Figure 2. Validation of Grin2a downregulation in the BTBR mouse hippocampus.

Supplementary Figure 1. The figure legends are required to have the same font as the main text, 12 point normal Times New Roman, single spaced. Please use a single paragraph for each legend and prepare the figures keeping in mind the PDF layout.
